# Trauma unit admissions at the Ugandan National Referral Hospital: a descriptive study

**DOI:** 10.4314/ahs.v22i1.49

**Published:** 2022-03

**Authors:** Tonny Stone Luggya, Annet Alenyo Ngabirano, Richardson Sarah, Jackie Mabweijano, John Osire, Lilian Achieng, Josephine Nabulime, Alex Bangirana

**Affiliations:** 1 Department of Anaesthesia and Critical Care, College of Health Sciences, Makerere University; 2 Department of Emergency Medical Services, Ministry of Health Uganda; 3 Emergency Medicine Department, University of Edinburgh; 4 Accident and Emergency Department, Directorate of Surgical services, Mulago Hospital

**Keywords:** Emergency care, Trauma, Uganda, Traumatic Brain Injuries, Accidents, Injury

## Abstract

**Background:**

Injuries are a neglected epidemic globally accounting for 9% global deaths; 1.7 times that of HIV, TB and malaria combined. Trauma remains overlooked with key research and data focusing on infectious diseases yet Uganda has one of the highest rates of traumatic injury. We described demographics of patients admitted to Mulago Hospital's Shock Trauma Unit within the Emergency Department.

**Methods:**

This was a retrospective record review Trauma Unit admissions from July 2012 to December 2015. Information collected included: age, sex, time of admission, indication for admission and mechanism of trauma.

**Results:**

834 patient records were reviewed. The predominant age group was 18–35 with majority of patients being male. 54% of patients presented during daytime with 46% admitted in the evening hours or overnight. Mechanism of injury was documented in 484 cases. The most common mechanism was Road Traffic Accident (67.4%), followed by assault (12.8%) and mob violence (5.6%). The most common indication for admission was traumatic brain injury (84.5%), followed by haemodynamic instability (20.0%) and blunt chest injury (6.1%).

**Conclusion:**

There's a significant burden of high-acuity injury particularly among males with RTAs as the leading cause of admission associated with Traumatic Brain Injury as main admission indication.

## Background

Injuries are a neglected epidemic worldwide1, accounting for 9% of deaths globally (1.7 times that of HIV, TB and malaria combined). 90% of these deaths occur in Low and Middle Income Countries (LMICs)^2^. With a projected 40% increase in global deaths due to injury, the WHO predicts that just one type of injuries, road traffic accidents, will rise from the 9th to the 3^rd^ highest cause of world burden of disease by 2030^2^.

Despite the magnitude of trauma within LMICs being recognized as reducing life-expectancy, it remains overlooked at an international level, with much of the research and data focus remaining on infectious diseases, malnutrition and non-communicable diseases^3^.

Uganda is a low-income country in sub-Saharan Africa. With a rapidly developing economy, increases in human and vehicular populations combined with an inadequate road infrastructure, poor enforcement of traffic regulations and inferior vehicle quality contribute to a high rate of casualties from road traffic crashes. The reported number of fatal crashes in Uganda has increased 7-fold in 25 years, from 500 in 1991 to 3,503 in 2016 according to annual Police Report Data^4^.

However, the WHO estimates the annual road fatality rate (reported and unreported) to be much higher at 12,0365, translating to a road traffic death rate of 29 per 100,000 population annually. In addition to this concerning rate of fatality, a further 12,754 serious injuries from crashes were reported in 2013^4^, with the actual rate unknown but likely to be significantly higher if all injuries were formally recorded.

The overall annual cost to the health system and the economy of road crashes is currently estimated at approximately $1.2 billion, equivalent to 5% of Uganda's gross domestic product (GDP)^4^. This figure relates only to road traffic related injury, the economic and health impact of injury of other causes such as assault and falls remains unknown.

Kampala is the capital city of Uganda and as result experiences a large burden of trauma related morbidity and mortality6. Mulago National Referral and Teaching Hospital (MNRTH), is Uganda's National tertiary referral hospital, faced with significant resource constraints and overwhelming service delivery challenges. Despite being the main center for trauma referrals for the country, the true extent of the injury burden at the facility is largely unknown, with a lack of accurate data available related to the actual utilization of the health services provided.

If health policy makers are to address the critical burden of injury in Uganda, they must first understand the epidemiology of patients presenting to health services following trauma. This study therefore aims to retrospectively determine the demographics of admissions to the Shock Trauma Unit in the Accident and Emergency Department of Mulago National Referral Hospital.

## Materials and methods

### Study Setting

MNRTH is a 1500 bed hospital offering specialised services and caring for approximately 140,000 patients annually. An average of 48,000 patients receive care through the Accident and Emergency Department each year with trauma related injuries, many of which are referred from Regional Centers throughout the country. The Shock Trauma Unit is comprised of 6 beds, 2 capable of delivering short-term mechanical ventilation (level 3 care) and 4 capable of High Dependency care delivery (level 2 care). The Unit is supported technically by the Department of Anaesthesia, staffed with 2 Specialist Doctors (1 Surgeon and 1 Anaesthesiologist) and 10 nurses, with an average nurse to patient ratio of 1:4. The Shock Trauma Unit receives patients from the A&E Department that require immediate critical level care as a temporising measure to formal ICU care, theatre procedures or where additional specialist input is required prior to ward admission.

### Ethical Approval

Ethical approval was obtained from the Department of Anaesthesia and Critical Care, Makerere University School of Medicine Internal Review Board and MNRTH IRB to conduct a 3-year retrospective and descriptive study performed from November 2015–16.

### Study Periods

Retrospective patient records and charts were collected from the period of the Trauma Unit creation in July 2012 to the end of December 2015.

### Study variables

The main variables collected on admission were referral source, demographic details, time of admission, indication of admission and type of trauma.

### Data management and analysis

A structured pretested and validated questionnaire was used as the data collection tool. All patient records were eligible for inclusion in the data set, with no exclusion criteria being applied other than lack of a patient record being available for analysis.

Confidentiality was observed by allocating unique study numbers for each patient to allow de-idenfying and anonmy sing of data at the point of collection. Once collected, data was compiled into a database using Epi DATA and statistical analysis performed using Prism version 8.0.

## Results

Basic descriptive statistics were used to analyze demographics data of eight hundred and thirty four (834) available patient records.

### Demographics

Of the 834 patient admission records, 86% were male and 14% were female, with 5 (0.5%) having no gender undocumented.

**Figure 1:**
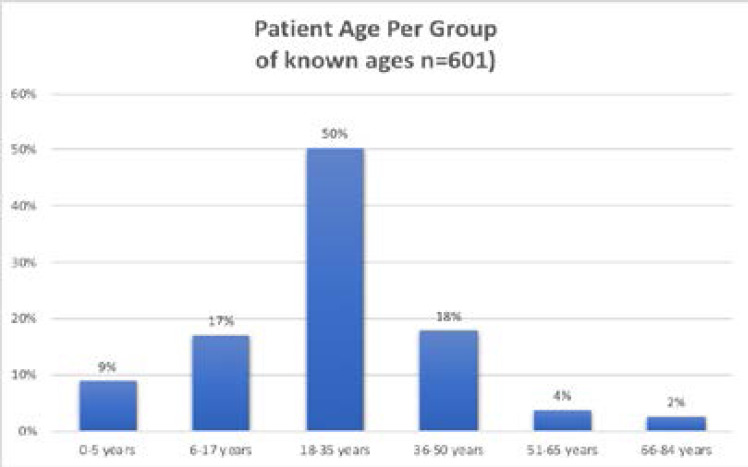
Breakdown of patient age in the ‘known’ age group

Age was documented in 601 out of 834 patients (72%). Of those with a documented age (n=601), 50% were between 18–35, with 17% aged 6–17 and 9% under 5. Those with undocumented age were 28% (n=233). Age ranged from 0–84 years. See figure 1.

### Admission Times

Admission time was documented in 592 cases, it was unknown in 242 cases. Documented admission times showed most patients 54% (n=317) presented at daytime, 24% (n=133) cases came at night and 22% (133) came in the evening.

### Mechanism of Trauma and Reason for Admission

**Figure 2:**
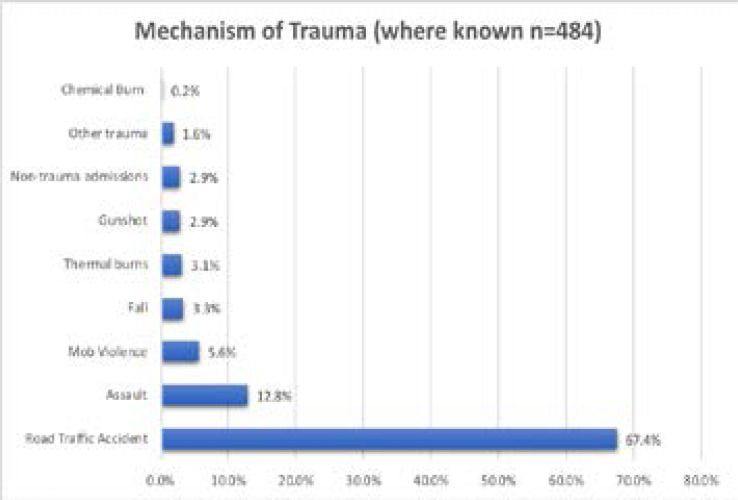
Mechanism of Trauma in ‘known’ group

The mechanism of trauma was recorded in 484 cases (unknown in 350 cases). Road Traffic Accident was the most common cause (67.4%), followed by assault (12.8%) and mob violence (5.6%). Of the known mechanisms of injury, non-trauma illnesses were documented for 2.9% of admissions. See Figure 2.

**Figure 3:**
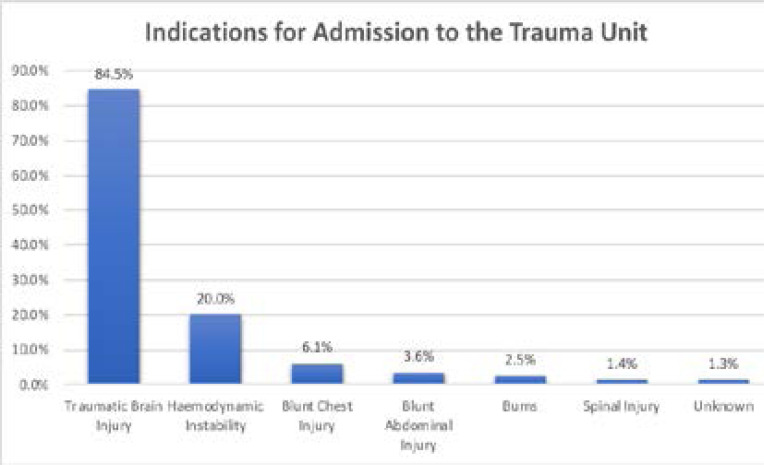
Indications for Admission to the Trauma Unit. Where multiple criteria, patient case represented in multiple groups (NB total >100%).

Patients were admitted to the Trauma Unit according to standard criteria: spinal injury, traumatic brain injury (TBI), chest injury, abdominal injury, hemodynamic instability and burns. 74.6% of patients fulfilled 1 of these criteria, with 20.2% fulfilling more than one criteria (2, 3 or 4 criteria). Of those admitted TBI was the most common indication identified in 84.5% of cases. Other criteria can be found in Figure 3.

## Discussion

By 2030 injury will be one of the top 5 the leading causes of death globally^7^. This three-year retrospective study is the first of its kind, analyzing the demographics of injury patients admitted to the MNRTH shock trauma unit, which receives the highest acuity level of patients in the emergency department. The data presented provides a true understanding of the burden of severe trauma patients being received at the national referral facility in Uganda.

The Emergency Care System in Uganda remains in its infancy, though it has increasingly become the focus of the Ministry of Health, Non-Governmental Organizations and the international health community^6^, ^8^, ^9^. For policy makers to make strategic implementation strategies that are contextualized to actual need and utilization of the system, data regarding injury and its impact on morbidity and mortality in the population is vital^10^.

Our study demonstrated that Road Traffic Accidents were the leading cause of severe injury requiring admission to the shock trauma unit, with TBI being the most common indication for admission. This finding is similar to those of other local^11^ and regional studies^12^. Traumatic brain injury is a critical problem for Uganda and its population. As represented by this study, the burden of this condition is significant with a large proportion of high-acuity trauma patients suffering TBI. Recovery from TBI in resource-limited context presents significant challenges^13^, as much of the care required is highly specialist, with ICU level intervention, neurosurgery and specific rehabilitation strategies required to achieve optimum outcomes for patients^14^. This advanced level of care is extremely costly and with the potential for good outcomes often limited due to injury severity, further research is required into the most effective way to manage TBI within the very resource-limited, high patient volume setting such as MNRTH.

The majority of patient cases were of working age (18–50 years), likely resulting in a significant economic burden to their dependents and their families, which is in line with findings in similar studies^15^, ^16^. The majority of patients were also male and therefore likely to be a key or main breadwinner for the family. The economic impact to the country, health system, families and individuals of trauma in Uganda remains largely unknown but requires further study if they country is to measure the true extent of investment required in the Emergency Medical System.

As is noted in other studies, a large number of patients were lacking all demographic details. This is common in clinical practice, where details are not recorded and the patient is registered as ‘unknown’. However local studies performed have shown that lack of supplies, overwhelming number of patients, and inadequate staffing also interfere with consistent monitoring and documentation of patients records^17^.

There is increasing evidence that implementing a robust trauma registry and injury surveillance system facilitates evidence-based resource allocation^18^. At present MNRTH is yet to commit to a formal injury surveillance system, which unfortunately continues to result in on-going poor resource allocation with significant bottle necks in service delivery^19^, confounded by overwhelming by patient numbers^20^. Only once the true utilization of the emergency trauma service is fully understood, through data capture and analysis, can policy makers address critical issues and adequately resource the Ugandan National facility to a level capable of reducing the morbidity and mortality experienced by its population.

## Conclusion

RTAs were the leading cause of admission to MNRTH Trauma unit with associated Traumatic Brain Injury as the main indication of admission. There was a paucity of complete and detailed patient data input on analysis of admission records. Further research is required into the effective management of trauma patients in low-resource settings, in addition to understanding the economic burden of trauma to individuals, the health system and the country.

## List of abbreviations

MNRTH: Mulago National Referral and Teaching Hospital.

A&E: Accident and Emergency.

RTAs: Road Traffic Accidents.

BI: Traumatic Brain Injury.

## Limitations

The study was a retrospective with issues of missing data that the research team couldn't locate because of time passed. This study finding was an issue of concern on its impact on complete analysis however the data review did give us a starting point to highlight important issues and identify opportunities for improvement.
